# HIV acute infection and long-term undisclosed HIV status among blood donors from the highly endemic Amazonas state, located in the Brazilian Amazon

**DOI:** 10.1016/j.bjid.2024.103848

**Published:** 2024-07-17

**Authors:** Maria Ivaldete Siqueira de Souza, Myuki Alfaia Esashika Crispim, Nelson Abrahim Fraiji, Alexander Leonardo Silva-Junior, Mariane Martins de Araújo Stefani

**Affiliations:** a Fundação Hospitalar de Hematologia e Hemoterapia do Amazonas (HEMOAM), Manaus, AM, Brazil; b Universidade Federal de Goiás, Instituto de Patologia Tropical e Saúde Pública, Goiânia, GO, Brazil

**Keywords:** HIV blood donors, HIV incidence, Brazilian Amazon

## Abstract

**Background:**

The Amazonas state/AM and Manaus rank among the highest AIDS detection rates in Brazil. High proportion of HIV infected blood donors and transmission clusters of multidrug antiretroviral/ARV resistant viruses were described in HEMOAM blood donors, a main Amazonas public blood bank. Recent and long-term infections among previously genotyped donors are reported.

**Methods/materials:**

The recency immunoassay Lag Avidity EIA (Maxim, USA) was employed. Clinical/CD4/viral load medical file data of the main local HIV management center (FMT-HVD) and ARV treatment/ART data were reviewed.

**Results:**

Among 142 HIV-blood donors, chronic infection predominated (*n* = 87; 61.3 %), 79 based on LAg EIA and 8 undisclosed HIV identified in FMT-HVD records, mostly young adult, single males, 4 repeat donors, all ART-naive. Recent infections represented 30.3 % (*n* = 43), 39 identified by LAg EIA and 4 immunologic windows (antibody negative/NAT/RNA positive). The overall profile of recent and long-term infections was similar, including moderate rate of transmitted drug resistance/TDR, however with multiple resistance mutations to more than one ARV-class, suggesting ART/failure.

**Discussion:**

Recent/acute and undisclosed/long-term HIV infections represent blood safety alerts suggesting test-seeking behavior of at-risk populations. Early ART use in Brazil, can turn HIV diagnosis more challenging representing a blood transfusion risk in the highly endemic Brazilian Amazon.

In the last decade, the Brazilian AIDS epidemic has declined especially in the Southeast and South regions, contrasting with the consistent increase observed in the North region.^1^^,^^2^ In 2021‒2022 the Amazonas state/AM, located in the North region, ranked the first and the second highest national AIDS detection rates, and Manaus, its capital, has reported detection rates much higher than the national rate.^1^^,^^2^ During the last decade, the Amazonas state reported the highest AIDS incidence rate (52.2/100,000 inhabitants) compared to the rate reported in Brazil (21.3/100,000).^2^ These recent epidemiologic data highlight the importance of surveillance and prevention measures for HIV/AIDS in Amazonas and Manaus, located in the Brazilian Amazon.

The importance of apparently healthy blood donors as a key sentinel population to monitor infectious diseases, including HIV is well known. Studies from our group have shown high rates of HIV-1 infection among HEMOAM blood donors, a reference public blood bank located in Manaus/AM.^3^^,^^4^ More recently, we have described HIV-1 molecular features including genetic subtypes and antiretroviral Transmitted Drug Resistance mutations (TDR) among 227 HIV infected blood donors from three reference public blood centers in North Brazil, the great majority (*n* = 198) was from HEMOAM.^5^, ^6^, ^7^, ^8^ Molecular epidemiology results of the HIV POL gene of blood donors from HEMOAM showed a strong predominance of subtype B (90 %) with minor circulation of subtypes C, F1 and BF1 recombinants.^5^ Additionally, moderate TDR rate (12.6 %) including diverse profiles of multiple drug resistance mutations and the identification of transmission clusters of multidrug-resistant viruses were identified.^6^ These findings emphasize the relevance of special surveillance efforts of the HIV/AIDS epidemic in Amazonas state, including incidence studies in specific populations, such as blood donors and the monitoring of TDR rates, which are crucial to orient treatment options and to assess proper interventions to control the epidemic.

HIV incidence assays, also known as recency assays, can provide incidence estimates by determining recent versus non-recent infections, however, their use is restricted to research and surveillance purposes. These assays are based on biomarkers such as antibody avidity, which is a function of the maturation of the immune response overtime. The limiting antigen Avidity Enzyme Immunoassay/EIA LAg was endorsed following the evaluation of seven HIV recency assays using a well-characterized panel of samples from the Consortium for the Evaluation and Performance of HIV Incidence.^9^ Thus the Centers for Disease Control and Prevention (USA) developed a LAg assay and transferred the technology to two biotech companies (Sedia and Maxim).^10^ A recent systematic review of limiting antigen avidity enzyme immunoassays for the detection of recent HIV infections reported that these tests can be considered valuable to incidence estimation, although different test features can influence results, such as HIV-1 subtypes, population characteristics, assay algorithms and thresholds.^11^

The goal of this study was to describe recent and long-term infections among previously genotyped HIV-1 infected blood donors from HEMOAM/AM based on Lag assay and reviews of medical file data and online data from the national antiretroviral treatment (ART) platform.^5^^,^^6^

Our study population was based on the original 198 HIV-HEMOAM blood donors described^5^^,^^6^ from which 142 stored plasma samples were available for testing. The recency LAg Avidity EIA test (Maxim Biomedical Inc., Rockville, MD, USA) was employed for incidence estimation according to manufacturer's instruction.^12^ Briefly, serum dilutions were incubated in 96-well micro titer plates coated with limiting concentration of gp41 (rIDR-M), a multi-subtype recombinant HIV-1 antigen. Low PH dissociation buffer was added, followed by a goat anti-human IgG conjugated to Horseradish Peroxidase (HRP). Finally, tetra methyl benzidine substrate was added and color generated. The optical density (OD = 450 nm) measured for each sample was normalized using a calibrator tested in triplicate in each plate, and the median of the three ODs was used to normalize specimen readings, producing normalized Optical Density (ODn) measurements. Specimens with ODn > 2.0 were considered long-term infections. Specimens producing an initial “screening” ODn ≤ 2.0 were subjected to triplicate “confirmatory” testing and the median ODn of the triplicate results was the final result. Recent infections were defined by ODn values ≤ 1.5. Additionally, clinical information, CD4 counts and viral load data were retrieved from medical files at Fundação de Medicina Tropical, Heitor Vieira Dourado (FMT-HVD), the main public reference center for HIV management located in Manaus/AM. ARV treatment/ART data registered at the national online HIV treatment platform of the Ministry of Health (SICLOM) was also revised.

In this study, acute infections were defined by the blood bank results of serologic immunologic window as HIV seronegativity and HIV Nucleic Acid Test (NAT) positivity, indicating early infection before seroconversion. HEMOAM implemented NAT ‒ screened donations to decrease the residual risk of immunologic window period transmission in 2012.^4^ Recent HIV infection was defined by the use Maxim LAg-Avidity EIA, that applies only to seropositive individuals and is based on the avidity of HIV antibodies, which correlates with infection duration. We have adopted the timing of recent infection indicated by the manufacturing company: 161 days (95 % CI 148‒174) at the cutoff ODn ≤1.5. Long-term infection (>161 days) was defined by Lag Avidity assay results and by data retrieved from medical files at FMT-HVD.

Statistical analysis was performed with absolute parameter values. Fisher's exact test was applied for comparisons of sociodemographic parameters among recent and long-term infections, *p* < 0.05 was considered significant. Medians and Interquartile Range (IQR) were applied to CD4 counts and viral load measurements. The Institutional Review Board approved this study (CAEE # 26904819.0000.0009).

Out of 142 blood-donor samples available, 12 were excluded from LAg testing due to previous knowledge of the timing of infection: four acute cases diagnosed as serologic immunologic window at HEMOAM^6^ and 8 long-term undisclosed cases of HIV infection previously diagnosed at FMT-HVD.

LAg avidity EIA results from 130 HIV donors (142–12 exclusions) showed that 79 were considered long-term and 39 were classified as recent infections; 12 specimens (8.4 %) had indeterminate results. Considering also data retrieved from medical files, long-term infections prevailed in HEMOAM HIV-1 infected blood donors (61.3 %, *n* = 87): 79 based on LAg EIA and 8 based on data from FMT-HVD medical files. Recent HIV-1 infections represented 30.3 % (*n* = 43), 39 identified by LAg EIA and 4 acute serologic immunologic window cases (Antibody negative/NAT positive)^6^ identified by the HEMOAM screening.

Similar sociodemographic and molecular profiles were observed in recent × long-term HIV infected HEMOAM blood donors: the majority was young (18‒30 years), males, singles, with high school degree (Table 1). Repeat donors were 51 % in recent infections and 38 % in long-term infections. Recent and long-term infections had high proportions of subtype B, BF1 recombinants and subtype F, while subtype C was only detected in long-term infections. According to previous genotyping data,^6^ the rates of transmitted drug resistance mutations in recent and long-term cases were comparable (13.9 % and 13.8 % respectively).

**Table 1:** Main sociodemographic and molecular features of recent and long-term HIV-1 infected HEMOAM blood donors.

Variable	Recent Infections (*n* = 43)		Long-term Infections (*n* = 87)		Total		*p*-value*
	n	(%)	n	(%)	n	(%)	
**Age (years)**							0.247
18‒30	31	(72)	51	(59)	82	(63)	
31‒50	12	(28)	32	(37)	44	(34)	
> 50	0	(0)	4	(4)	4	(3)	
**Gender**							0.772
Male	39	(91)	76	(87)	115	(88)	
Female	4	(9)	11	(13)	15	(12)	
**Schooling**							0.935
Elementary	5	(12)	12	(14)	17	(13)	
High School	32	(74)	62	(71)	94	(72)	
University	4	(9)	10	(11)	14	(11)	
NA	2	(5)	3	(3)	5	(4)	
**Marital status**							0.232
Single	38	(88)	69	(79)	107	(82)	
Married/Stable union	5	(12)	18	(21)	23	(18)	
**Donor Type**							0.187
First time	21	(49)	54	(62)	75	(58)	
Repeat	22	(51)	33	(38)	55	(42)	
**HIV-1 subtype**							0.449
B	40	(93)	73	(84)	113	(87)	
F	1	(2)	2	(2)	3	(2)	
C	0	(0)	4	(5)	4	(3)	
BF1	2	(5)	8	(9)	10	(8)	
**ARV Resistance**							0.169
NRTI	1	(2)	8	(9)	9	(7)	
NNRTI	6	(14)	10	(11)	16	(12)	
PI	1	(2)	0	(0)	1	(0.8)	

ARV, Antiretroviral Resistance Mutations; NRTI, Nucleoside Reverse Transcriptase Inhibitor mutations; NNRTI, Non-Nucleoside Reverse Transcriptase Inhibitor mutations; PI, Protease Inhibitor mutations; NA, Not Available.

The four acute HIV infections detected in blood donors were young males, and one had a POL sequence (GenBank#MH6731121) that belonged to a transmission cluster of multidrug resistance virus (Table 2). The review of medical files one year after genotyping studies were published^5^^,^^6^ showed that 61.9 % (88 out of 142) of infected donors were enrolled at the FMT-HVD, the majority (*n* = 80) was registered after the genotyping study. This review revealed that 5.6 % (8 out of 142) of donors investigated had a previous diagnosis of HIV infection and were therefore considered as long-term infections (Table 2). Among the 8 HIV undisclosed long-term infected donors, six were males, four were repeat donors, that had made from 1 to 11 blood donations at HEMOAM, all of them before the genotyping study (data not shown). These individuals were mostly young adults (22‒40 years), one had a POL sequence with multiple nucleoside and non-nucleoside reverse transcriptase inhibitor mutations (NRTI/NNRTI) (GenBank #MH673188.1) (Table 2). According to the review of the national online HIV treatment platform SICLOM, none of them was under ART at the time of the blood donation and the genotyping study.

**Table 2:** Main sociodemographic and molecular features of acute immunologic window and undisclosed long term HIV infected HEMOAM blood donors.

GenBank number	HIV Infection	Gender	Age (years)	Donor type	HIV-diagnosis (site/year)	TDR	HIV-1 Subtype (RT/PR)
MH673126.1	IW	M	27	FT	H/2015	No	BB
MH673112.1	IW	M	25	R	H/2013	NNRTI^a^	BB
MH673209.1	IW	M	24	R	H/2012	No	BB
MH673098.1	IW	M	21	FT	H/2013	No	BB
MH673188.1	LT	M	27	R	FMT/2015	NRTI+NNRTI^b^	BB
MH673089.1	LT	M	40	FT	FMT/2014	No	BB
MH673132.1	LT	M	34	R	FMT/2015	No	BB
MH673109.1	LT	M	22	FT	FMT/2013	No	BB
MH673258.1	LT	M	37	FT	FMT/2016	No	BB
MH673222.1	LT	F	33	FT	FMT/2016	No	CC
MH673186.1	LT	F	31	FT	FMT/2015	No	BB
MH673251.1	LT	M	29	R	FMT/2016	No	BB

IW, Serologic Immunologic Window cases (HIV-NAT+/Antibody-); LT, Long-Term infections: undisclosed HIV cases. M, Male; F, Female; R, Repeat donor; FT, First Time donor; H, HEMOAM, Manaus, AM; FMT-HVD, “Fundação de Medicina Tropical Heitor Vieira Dourado”, Manaus, AM; TDR, Transmitted Drug Resistance mutations; NRTI/NNRTI, Nucleoside and Non-Nucleoside Reverse Transcriptase Inhibitor resistance mutations.

a Sample belonging to a previously described multidrug resistant transmission cluster.^6^ #NNTRI mutations: E138A, V179D.

b NTRI mutations: M41L, E44D, D67N, T69D, L74I(V), L210W, T215D/ NNRTI mutations: K103N, V108I. RT/PR, Reverse Transcriptase/Protease Regions.

According to medical files review at FMT-HVD, the median of the first reported CD4 cell counts in the recent infected group (*n* = 24) was 488 cells/µL (IQR 346‒655 cells/μL) and the median in the long-term infected group (*n* = 51) was 349 cells/μL (IQR 176‒535 cells/μL). The median of the first reported Viral Load (VL) in the recent infected group (*n* = 22) was 16,066 copies/mL (IQR 2848‒71,526 copies/mL) and the median in the long-term infected group (*n* = 45) was 45,784 copies/mL (IQR 8871‒169,792 copies/mL. However, it's important to point out that the first measurements of these parameters were obtained at different time points post diagnosis, depending on how long each patient took to search for specialized assistance, thus limiting the value of the association of these parameters to the time since infection.

In the current study from the Brazilian Amazon, recent infections including serologic immunologic window cases and undisclosed long-term HIV infections were identified among apparently healthy HEMOAM blood donors, reflecting the highly endemic situation in Amazonas, one of the hottest spots for HIV-1 transmission in Brazil.^1^^,^^2^ Studies in different HIV infected Brazilian populations have applied diverse methodologies to distinguish recent from long-term infections. Two multicenter studies based on Lag avidity assay among blood donors reported HIV incidence among seropositive donors from different geographical Brazilian regions. A study among 246 HIV ‒ seropositive donors from 4 different blood centers identified 17.5 % as recent infection, a much lower estimate than the 30.3 % described in the current study.^13^ Another study analyzed 10 year period (2007‒2016) incidence in first time and repeat donors from blood centers located in distinct Brazilian regions: Recife, São Paulo and Belo Horizonte and during a shorter period in Rio de Janeiro. Although no blood center from north Brazil was included, this study showed that Recife in the northeast region reported the highest incidence estimates both in first time and repeat donors.^14^

In our study, undisclosed HIV represented almost 10 % of the long term-infections. Self-report is often used to identify HIV-infected individuals who are not aware of their HIV status, however, the accuracy of this information is limited, as infected individuals may deny their HIV status,^15^ similarly to what occurred during the blood bank interview. Undisclosed HIV status may be associated with concern about the confidentiality of the information, or with fear of stigma, discrimination, exclusion from study benefits or interventions, or of other social harms.^16^ Although the current study was not designed to identify blood donation motivation, we can speculate test-seeking behavior especially in undisclosed HIV cases, as the blood bank screening was probably used to ratify the HIV diagnosis. Additionally, the finding of one third of HIV infected blood donors with recent infection, including serologic immunologic window cases, which can be considered early cases before seroconversion, corroborates the test-seeking hypothesis of at risk donors, who take advantage of the blood bank screening to monitor their HIV-1 status. This finding raises important blood safety concerns, as it's known that high viral loads during the early HIV infection increase the risk of transmission by blood transfusion. In this context, our results reassure the effective role of the HIV-NAT as an important additional safety layer to avoid the residual risk of transmission by blood transfusion, especially among serologic immunologic window cases.

Studies have used retrospective ARV testing in samples as an objective measure to evaluate the accuracy of self-reported HIV status.^17^^,^^18^ A study in South African blood donors to enroll HIV elite controllers/EC (Antibody+/RNA-), found upon ARV drugs testing, that almost 70 % of presumed EC were in fact previously diagnosed HIV under ART.^18^ In our study, the resistance profiles of HEMOAM blood donors, characterized by the simultaneous detection of multiple ARV resistance mutations, associated to NRTI/NNRTI^6^ suggest ART and failure, however medical files and SICLOM platform revisions could not confirm ART and ARV drugs testing was not available.

Studies have shown that early ARV use may reduce both HIV-RNA and antibody levels^19^^,^^20^ increasing the difficulties to identify cases of HIV infection among blood donors with undisclosed HIV and under ART. Also, it's possible that effective ART and undetectable viral loads may be misinterpreted by patients as “cure” motivating blood donation to check results by a most reliable serologic and molecular blood bank criterion. Although we cannot estimate the extent of undisclosed HIV and ART among HEMOAM blood donors, HIV positivity and concomitant early ARV use (HIV+/ARV+) may represent a new challenge for the local blood supply safety as recent epidemiologic data showed that the Amazonas has one of the highest AIDS incidence rates in Brazil.^1^^,^^2^

In summary our findings of both undisclosed long-term and of recent HIV infections including serologic immunologic window cases among apparently healthy blood donors, some of them repeat donors, represent an alert for blood bank safety in Amazonas state. Considering the complex TDR profiles detected^6^ and the widespread early use of ART and pre/post exposure ARV prophylaxis recommended by the Brazilian Ministry of Health, new blood bank safety strategies may be needed, as both viral load and antibody production can be reduced and become negative upon early ART. This possibility raises crucial public health blood transfusion concerns, especially in highly endemic areas as the Amazonas state, Brazil.

## Funding

“Fundação de Amparo à Pesquisa do Estado do Amazonas: CAPES/FAPEAM (EDITAL 24/2014) and (PVN-II, PROESTADO 002/2008,007/2018 and 005/2019). MMAS is a research fellow from FAPEAM PVN-II, (PECTI-AM/SAÚDE Program, Grant #004/2020) and from the Brazilian Research Council/ CNPq Grant # 311986-2019-6). ALSJ is a PhD student from PPGBIOTEC, UFAM, Manaus, AM.

## Conflicts of interest

The authors certify that they have no financial or non-financial interest in the subject or materials discussed in this manuscript.
